# Reclassification of Pasteurella skyensis as Phocoenobacter skyensis comb. nov. and description of Phocoenobacter atlanticus sp. nov. isolated from diseased Atlantic salmon (Salmo salar) and lumpfish (Cyclopterus lumpus), with subdivision into Phocoenobacter atlanticus subspecies atlanticus subsp. nov. and Phocoenobacter atlanticus subspecies cyclopteri subsp. nov.

**DOI:** 10.1099/ijsem.0.006729

**Published:** 2025-04-09

**Authors:** Hanne K. Nilsen, Anne Berit Olsen, Thomas H. Birkbeck, Farah Manji, Duncan J. Colquhoun, Snorre Gulla

**Affiliations:** 1 Section of Aquatic Biosecurity Research, Norwegian Veterinary Institute, Bergen, Norway; 2 Division of Infection and Immunity, University of Glasgow, Glasgow, Scotland, UK; 3 Mowi Norway, Bergen, Norway; 4 Fish Health Research Group, Norwegian Veterinary Institute, Ås, Norway

**Keywords:** Atlantic salmon (*Salmo salar*), fish disease, lumpfish (*Cyclopterus lumpus*), *Pasteurella*, *Pasteurellaceae*, *Phocoenobacter*

## Abstract

*A corrigendum of this article has been published full details can be found at* *https://doi.org/10.1099/ijsem.0.006788*.

Gram-negative bacteria, similar in 16S rRNA gene sequence to *Pasteurella skyensis*, have been irregularly cultured from diseased Atlantic salmon (*Salmo salar*) sea-farmed in Norway since the 1980s and more commonly in later years from diseased lumpfish (*Cyclopterus lumpus*) farmed in the northeast Atlantic area. Phenotypic and genetic analyses confirmed that the majority of Norwegian isolates in the present study, cultured from both salmon and lumpfish, are closely related to, yet distinct from, *P. skyensis* and represent a hitherto undescribed species. We further argue, based on genetic similarity levels, that the original placement of *P. skyensis* within *Pasteurella* was erroneous and that this species belongs within *Phocoenobacter*, as *Phocoenobacter skyensis* comb. nov. Accordingly, we propose the name *Phocoenobacter atlanticus* sp. nov. for the new species characterized here. Furthermore, this species separates into two genetically and phenotypically distinct lineages, respectively, associated with Atlantic salmon and lumpfish, and for which we propose the names *Ph. atlanticus* subsp. *atlanticus* subsp. nov. and *Ph. atlanticus* subsp. *cyclopteri* subsp. nov., respectively. The type strain for *Ph. atlanticus* subsp. *atlanticus* subsp. nov. is strain NVIB3624^T^ (NCIMB 15512^T^, CCUG 77520^T^), and the type strain for *Ph. atlanticus* subsp. *cyclopteri* subsp. nov. is strain NVIO9100^T^ (NCIMB 15511^T^, CCUG 77521^T^).

## Introduction

While most members of the family *Pasteurellaceae* are commensals, some are important pathogens of terrestrial animals, including humans [1]. With reclassification of *Pasteurella piscicida* [2] as *Photobacterium damselae* subsp. *piscicida* [3], *Pasteurella skyensis* [4] is currently the only validly published fish-pathogenic species within the *Pasteurellaceae*. *P. skyensis* is associated with significant, recurring mortalities in sea-farmed Atlantic salmon, *Salmo salar*, in Scottish aquaculture [5,6] and forms a monophyletic branch with *Phocoenobacter uteri* [7], isolated from the uterus of a stranded harbour porpoise, *Phocoena phocoena* [8]. *P. skyensis* was also recently identified in a localized outbreak in salmon farmed in Norway [9].

Outbreaks of disease associated with isolates phenotypically and genetically similar, but not identical, to *P. skyensis* [4] have been increasingly diagnosed in recent years in Norwegian farmed salmon and in farmed lumpfish, *Cyclopterus lumpus*, in both Norway and Scotland [7,10]. The disease associated with infections in salmon, first identified in the late 1980s, was then termed ‘varracalbmi’ (‘blood-eye’ in the Sámi language [11]). A previous 16S rRNA – and *rpoB* gene – sequence-based phylogenetic study clearly separated Norwegian isolates from *P. skyensis* [10], while a more recent genome-based study highlighted the existence of a highly conserved lumpfish-specific lineage and a more diverse salmon-specific lineage [7].

Here, using a polyphasic approach, we propose reclassification of *P. skyensis* as *Phocoenobacter skyensis* comb. nov. and establishment of a new species, *Phocoenobacter atlanticus* sp. nov., within *Phocoenobacter*. *Ph. atlanticus* sp. nov. is further divided into the two host-specific subspecies *atlanticus* subsp. nov. and subspecies *cyclopteri* subsp. nov.

## Isolation and ecology

Details of the isolates studied, collected from Atlantic salmon in Norway (*n*=11) and Scotland (*n*=4) and lumpfish in Norway (*n*=10), between 1991 and 2020, are shown in Table 1. The type strain of *Ph. uteri*, NCTC 12872^T^ isolated from a harbour porpoise, *Pho. phocoena*, was also included. All fish isolates were cultured from either the kidney, heart, skin ulcer or eye of diseased Atlantic salmon or lumpfish farmed in seawater. Tissue samples were inoculated onto blood agar (5% bovine blood) with a final concentration of 2% NaCl (BAS) and incubated aerobically at 22 °C. After 2–5 days of incubation, variable numbers of small (≤1 mm), grey, initially non-haemolytic colonies were recovered in pure culture or as the dominating flora in mixed cultures. Pure subcultures were then maintained at –80 °C. Subcultures on BAS were incubated aerobically at 22 °C for 3–4 days to provide inocula for biochemical testing or DNA extraction.

**Table 1.** Isolates included in the study

Proposed species/subspecies	Isolate id.	Alternative id.	Isolated from	Year	Country	Latitude*	Longitude*	RefSeq acc. no.	Initial ref.
*Ph. atlanticus* subsp. *atlanticus*	NVIB3689	NVIO1993	*S. salar*	1991	Norway	69.649610	18.957010	GCF_030764075.1	[10]
*Ph. atlanticus* subsp. *atlanticus*	NVIB3131		*S. salar*	1999	Norway	60.200000	5.700000	GCF_030764485.1	[7]
*Ph. atlanticus* subsp. *atlanticus*	NVIB3694	NVIO8239	*S. salar*	2012	Norway	61.170833	5.293056	GCF_030764015.1	[10]
*Ph. atlanticus* subsp. *atlanticus*	NVIB2635		*S. salar*	2018	Norway	60.350000	5.000000	GCF_030764645.1	[7]
*Ph. atlanticus* subsp. *atlanticus*	NVIB3624^T^	NCIMB 15512^T^	*S. salar*	2019	Norway	61.599600	5.032800	GCF_030764225.1	[7]
*Ph. atlanticus* subsp. *atlanticus*	NVIB3687		*S. salar*	2019	Norway	62.471239	6.154239	GCF_030764125.1	[7]
*Ph. atlanticus* subsp. *atlanticus*	NVIB3642		*S. salar*	2019	Norway	61.808611	5.420833	GCF_030764205.1	[7]
*Ph. atlanticus* subsp. *atlanticus*	NVIB3672		*S. salar*	2019	Norway	60.370000	6.143889	GCF_030764105.1	[7]
*Ph. atlanticus* subsp. *atlanticus*	NVIB3763		*S. salar*	2020	Norway	60.350000	5.000000	GCF_030763825.1	[7]
*Ph. atlanticus* subsp. *atlanticus*	NVIB3708		*S. salar*	2020	Norway	60.037778	5.268333	GCF_030763925.1	[7]
*Ph. atlanticus* subsp. *cyclopteri*	NVIO3648		*C. lumpus*	1996	Norway	69.649610	18.957010	GCF_030764525.1	[7]
*Ph. atlanticus* subsp. *cyclopteri*	NVIB164		*C. lumpus*	2012	Norway	58.970000	5.731389	GCF_018343795.1	[7]
*Ph. atlanticus* subsp. *cyclopteri*	NVIB3802		*C. lumpus*	2013	Norway	68.221389	13.784444	GCF_030763505.1	[7]
*Ph. atlanticus* subsp. *cyclopteri*	NVIB543	NVIO9294	*C. lumpus*	2013	Norway	58.970000	5.731389	GCF_030764665.1	[10]
*Ph. atlanticus* subsp. *cyclopteri*	NVIO9100^T^	NCIMB 15511^T^	*C. lumpus*	2013	Norway	62.567500	6.372222	GCF_018343795.1	[10]
*Ph. atlanticus* subsp. *cyclopteri*	NVIB1365		*C. lumpus*	2016	Norway	60.402778	5.805556	GCF_030764725.1	[7]
*Ph. atlanticus* subsp. *cyclopteri*	NVIB3801		*C. lumpus*	2017	Norway	69.300000	17.666667	GCF_030764785.1	[7]
*Ph. atlanticus* subsp. *cyclopteri*	NVIB1926		*C. lumpus*	2017	Norway	60.200000	5.700000	GCF_030763465.1	[7]
*Ph. atlanticus* subsp. *cyclopteri*	NVIB2702		*C. lumpus*	2018	Norway	60.200000	5.700000	GCF_030764685.1	[7]
*Ph. atlanticus* subsp. *cyclopteri*	NVIB2993		*C. lumpus*	2018	Norway	59.998333	5.577222	GCF_030763415.1	[7]
*Ph. skyensis*	95A1^T^	NCIMB 13593^T^	*S. salar*	1995	Scotland	56.659406	−4.011214	GCF_013377295.1	[4]
*Ph. skyensis*	01A1		*S. salar*	2001	Scotland	56.659406	−4.011214	GCF_030763365.1	[6]
*Ph. skyensis*	TW34_18		*S. salar*	2017	Scotland	56.659406	−4.011214	GCF_030764825.1	[7]
*Ph. skyensis*	TW260_19		*S. salar*	2019	Scotland	56.659406	−4.011214	GCF_030764885.1	[7]
*Ph. skyensis*	NVIO11850		*S. salar*	2020	Norway	60.066667	6.550000	GCF_030763395.1	[9]
*Ph. uteri*	NCTC 12872^T^	M1063U/93^T^	*Pho. phocoena*	1993	Scotland	56.659406	−4.011214	GCF_900454895.1	[8]

*Approximate coordinates (municipal administrative centre or midpoint) are provided in order to retain the anonymity of affected aquaculture sites in Norway. For Scottish isolates, available information on their geographic origins was limited.

## 16S rRNA gene sequences

Partial 16S rRNA gene sequences (generated by Sanger sequencing) for *Ph*. *atlanticus* type strains NVIB3624^T^ (subsp. *atlanticus*; 1,324 bp; present study) and NVIO9100^T^ (subsp. *cyclopteri*; 1,456 bp; previously published [10]) are available via NCBI GenBank under accession numbers OR265624 and KJ585696, respectively. For reciprocal authenticity validation of the two 16S rRNA gene sequences and their corresponding genomes (see below), alignments were performed with the NCBI online blastn tool, in both cases yielding 100% query coverage and identity.

## Genome features

For all 26 isolates assessed phenotypically in the present study, previously generated genome assemblies available via the NCBI RefSeq repository (see accession numbers in Table 1) were compiled for comparative analyses.

**Fig. 1.**
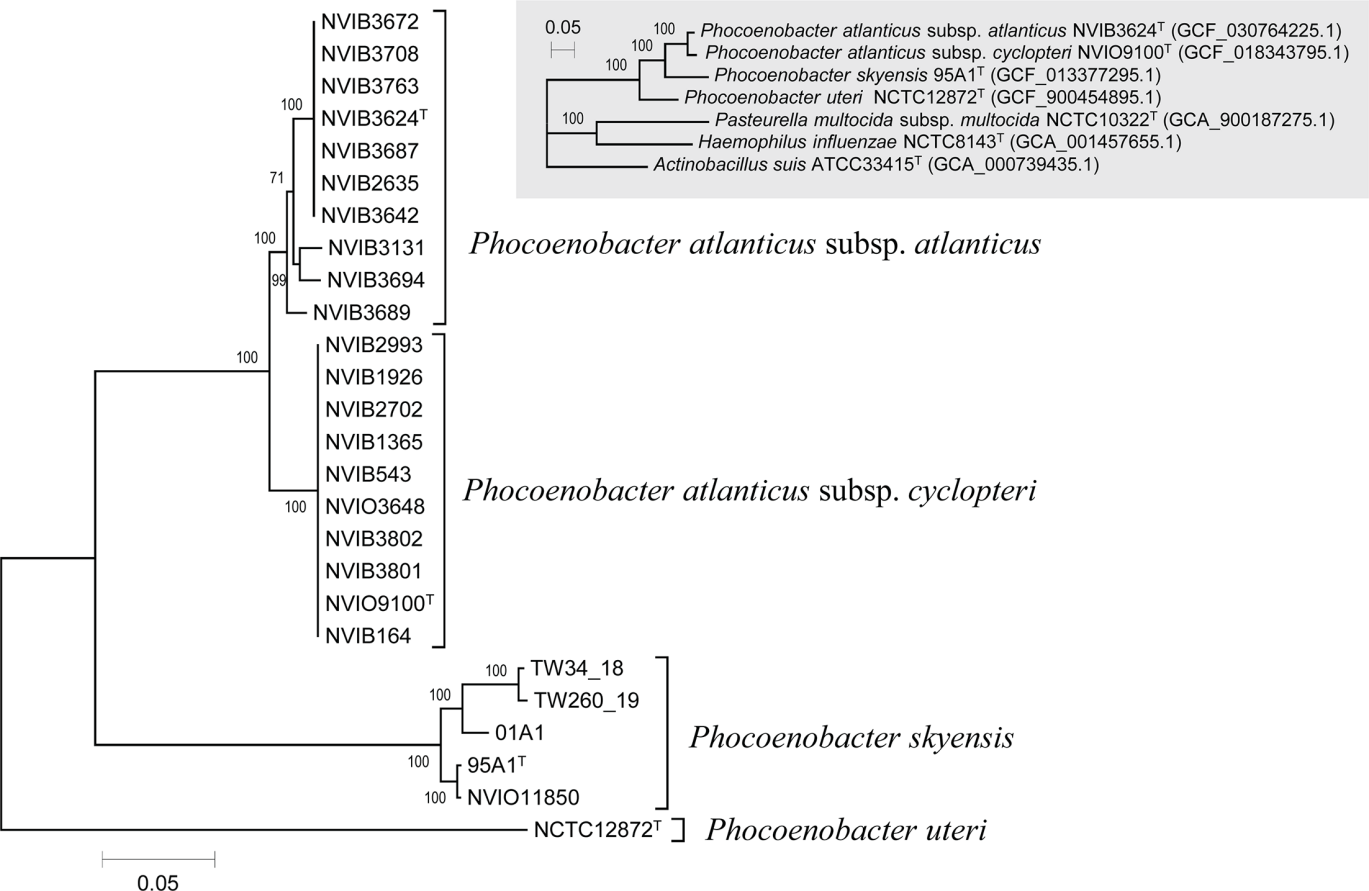
Maximum likelihood phylogenetic reconstruction for the studied isolates, inferred from a concatenated alignment of 989 core genes shared across all 26 genomes. Via the same approach (289 core genes), the inset tree on the top right showcases the monophyletic nature of the three *Phocoenobacter* species combined, by comparison towards other *Pasteurellaceae* representatives (only type strains included; accession numbers in parentheses). Scale bars refer to nucleotide substitutions per site, and bootstrap support values are shown at nodes. See the section on Genome features for further methodological details. Accession numbers are given in Table 1.

Following annotation of all 26 genomes with Prokka v1.14.5 [12], the core genome was inferred using Panaroo v1.3.4 [13], with clean mode set to ‘sensitive’, sequence identity and length difference thresholds both set to 85%, and core genome sample threshold set to 99%. From the 989 core genes thus identified, a 976,564 bp concatenated alignment was created in PRANK [14] and used for maximum likelihood phylogenetic reconstruction in IQ-TREE v2.1.4 [15,16]. ModelFinder [17] was employed across all GTR, HKY and SYM models, as well as ultrafast bootstrap branch support estimation [18] with 10,000 replicates. The resulting phylogenetic tree, visualized using MEGA v7.0.26 [19], clearly separates the studied isolates (with the exception of *Ph. uteri* NCTC 12872^T^) into two main monophyletic lineages, i.e. *Ph. skyensis* and *Ph. atlanticus* (Fig. 1). A separate inset tree is also shown, visualizing the monophyletic nature of the *Phocoenobacter* genus. This latter tree was made on the basis of type strain genomes from *Phocoenobacter* in addition to selected *sensu stricto* representatives of three other *Pasteurellaceae* genera [20]. For phylogenetic recreation, the same procedure as outlined above was employed, except for the sequence identity and length difference thresholds in Panaroo, which were lowered to 80%, resulting in a 288,608 bp alignment spanning 289 core genes.

**Fig. 2.**
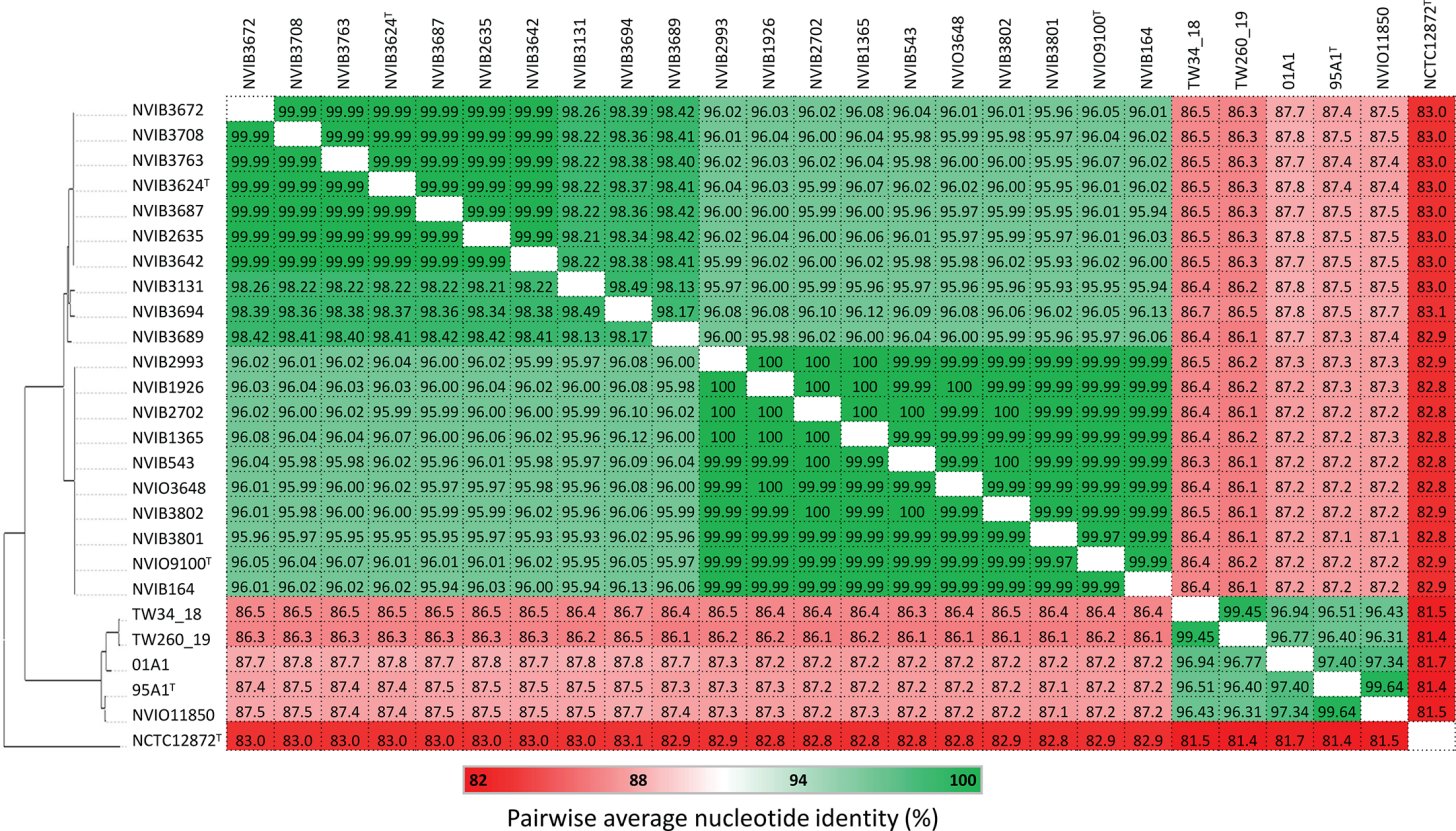
Pairwise ANI values between the studied genomes, with colours reflecting degrees of similarity (see reference bar).

Pairwise average nucleotide identities (ANI), assessed by FastANI v1.3 [21] with --fragLen set to 500 and --minFraction set to 0.1, further underpin that *Ph. skyensis* and *Ph. atlanticus* should be regarded as separate species, with inter-lineage ANI values of 86.1–87.8% (Fig. 2). This is significantly below the proposed prokaryotic species threshold [21,23]. Moreover, while maximum ANI disparity values of 95.93% within *Ph. atlanticus* suggest that this lineage represents a single, as yet undescribed, bacterial species, this analysis also reveals its separation into two main sub-lineages with maximum ANI disparity values of 99.97% (*Ph. atlanticus* subsp. *cyclopteri*) and 98.13% (*Ph. atlanticus* subsp. *atlanticus*), respectively.

Assembly evaluations using Quast v5.0.2 [24] on default settings revealed that the investigated *Ph. atlanticus* subsp. *atlanticus* (*n*=10) and subsp. *cyclopteri* (*n*=10) genomes range, respectively, in size between 2.12–2.29 and 2.25–2.30 Mbp, with G+C contents of 34.21–34.29 and 34.24–34.47 mol%. In comparison, the investigated *Ph. skyensis* (*n*=5) genomes display sizes between 2.16 and 2.27 Mbp and G+C contents of 34.86–34.95 mol%, while the single *Ph. uteri* genome has a size of 2.18 Mbp and a G+C content of 36.17 mol%. Overall, the 26 genome assemblies consist of 1–93 (median 54) contigs/scaffolds, with N50 ranging between 59 and 2,302 (median 101) kbp and L50 between 1 and 14 (median 8). For further details, see Table S1, available in the online Supplementary Material.

## Phenotypical features

All 26 isolates investigated were subjected to a comprehensive panel of phenotypic tests, all of which, unless otherwise stated, were performed in parallel at 22 °C.

Gram staining, cell morphology and motility were studied at 1,000× magnification with a Leica DMLS microscope. The ability to grow anaerobically was tested on BAS incubated for 7 days using the GENbox test system (bioMérieux). Catalase activity was determined using 3% (v/v) H_2_O_2_ on bacterial material transferred to a glass slide, and the presence of cytochrome oxidase and indole was evaluated using the BBL™ Dryslide oxidase and indole tests (Becton Dickinson), respectively.

Enhancement of growth by NaCl was tested by culture on bovine blood agar (5%) containing 0.5 and 2% NaCl. Dependence on blood for growth was tested on tryptic soy agar (TSA) with 2% NaCl (Oxoid). Dependence on serum components for growth was tested on TSA containing 2% NaCl, with and without bovine serum supplementation (3% v/v). Requirement for X- and V-factors was tested on TSA with 2% NaCl using X- and V-factor discs (Oxoid), with plates being read after 4 days of aerobic incubation.

Production of acid from various carbohydrate sources was investigated using a heavy inoculum method in BBL™ Phenol Red Broth Base (Becton Dickinson) supplemented with 10% foetal bovine serum (Biosera) and read after 4 days of incubation. The following carbohydrate sources were included: d(−) arabinose, d(+) cellobiose, d(+) mannose, d(+) sorbitol, d(+) xylose (Sigma), d(+) glucose, lactose, d(−) mannitol, trehalose (Merck) and d(+) maltose (Fluka). A colour change from red to yellow/bright orange was recorded as positive.

Susceptibility to the vibriostatic agent O129 (150 µg; Diatabs™ Rosco) was tested on BAS, and any zone of inhibition around the disc was regarded as evidence of susceptibility. Arginine dihydrolase, lysine decarboxylase and ornithine decarboxylase tests were carried out with Moeller decarboxylase medium (Becton Dickinson), supplemented with 3% bovine serum, and reactions were recorded after 7 days of incubation. The Voges–Proskauer (VP) reaction was examined using VP diagnostic tablets (Diatabs™ Rosco).

Beta-galactosidase and alpha-fucosidase production was assessed using the API ZYM (bioMérieux) system according to the manufacturer’s instructions with the exception of incubation temperature and duration (altered to 22 °C for 18–24 h) and the use of an isotonic 0.9% NaCl solution for preparation of inoculum.

Phenotypical characteristics discriminatory for the respective taxa described are listed in Table 2. Non-discriminatory characteristics are included in Table 2 legend.

**Table 2.** Differential phenotypical characteristics for: *Ph. atlanticus* subsp. *atlanticus* (1), *Ph. atlanticus* subsp. *cyclopteri* (2), *Ph. skyensis* (3) and *Ph. uteri* (4)

Characteristic	1 (*n*=10)	2 (*n*=10)	3 (*n*=5)	4 (*n*=1)
Growth at 37 °C	−	−	v	+
Growth at 4 °C	+	+	−	−
Growth on blood agar	+	w	+	+
Anaerobic growth	+	w	+	+
Haemolysis	−(w)^*γ*^	−(w)^*γ*^	−(w)^*γ*^	w^*ε*^
Catalase	−	−	−	+
Indole	−	−	+	−
Lysine decarboxylase	−	−	+	+
Ornithine decarboxylase	−	−	+	+
**Acid from**:				
d(−) Arabinose	+	−	v	−
d(−) Mannitol	+	−	v	−
d(+) Mannose	+	+	+	−
d(−) Sorbitol	+	−	+	−
Trehalose	+	−	v	−
d(+) Xylose	+	−	−	−
**API ZYM**				
*β*-Galactosidase	v	−	+	−
*α*-Fucosidase	−	+	v	−

Abbreviations: +, only positive reactions; −, only negative reactions; v, variable; w, extremely weak growth. All strains are positive for: growth at 30, 22 and 15 °C; growth on blood agar with end concentration of 2% NaCl and on TSA with 2% NaCl+serum; oxidase; VP; acid production from (d+) glucose, lactose and (d+) maltose; O129 (vibriostatic agent) sensitivity. All strains are negative for: growth on TSA with 2% NaCl; X/V factor requirement for growth; arginine dihydrolase degradation; acid production from (d+) cellobiose. For *Ph. uteri*, acid production and degradation tests were conducted at 30 °C. *γ* weakly *α*-haemolytic after extended cultivation. *ε* weakly *β*-haemolytic at 30 °C, in contrast to [8] tested at 37 °C CO_2_ atm.

## Discussion

The studied isolates are all in possession of phenotypical characteristics consistent with placement within the family *Pasteurellaceae*, although *Ph. atlanticus* subsp. *cyclopteri* grows extremely weakly under anaerobic conditions. All are non-motile, pleomorphic, Gram-negative rods and display weak cytochrome oxidase activity. With the exception of *Ph. uteri*, all lack catalase activity. The phenotypical differences for *Ph. uteri* reported here compared to those previously reported [8] are probably related to the higher concentrations of serum used in culture media in the present study. *P. skyensis* was originally proposed as a member of the genus *Pasteurella* based on a relatively high degree of 16S rRNA gene similarity with a bacterium then termed ‘*Pasteurella phocoenarum*’ [4]. However, 2 years prior, ‘*P. phocoenarum*’ had in fact been validly named as *Ph. uteri* [8]. The genetic analyses presented here clearly establish that the fish-pathogenic isolates studied belong within *Phocoenobacter*, clarify the taxonomic situation concerning the former *P. skyensis* and in turn expand *Phocoenobacter* from a single species to three, i.e. *Ph. uteri*, *Ph. skyensis* and *Ph. atlanticus*, with the latter further divided into the host-specific and phenotypically distinct subspecies *atlanticus* and *cyclopteri*. Recent work [7] involving whole-genome analysis of a larger number of geographically and chronologically disparate isolates revealed a degree of genetic variation within *Ph. skyensis* and *Ph. atlanticus* subsp. *atlanticus*. In contrast, *Ph. atlanticus* subsp. *cyclopteri* appears to be genetically highly conserved, possibly reflecting a strict parasitic relationship between this subspecies and its lumpfish host [7]. Identification of highly similar 16S rRNA gene sequences to those found in the *Phocoenobacter* spp. described here, from the microbiotas of various marine cetaceans, may indicate that these animals represent a natural reservoir for these types of bacteria [7]. It further suggests that this genus is likely to be supplemented with new species in the near future.

## Protologues

### Emended description of *Phocoenobacter* [8]

*Phocoenobacter* (Pho.coe.no.bac'ter. M.L. n. *phocoena* derived from Gr. n. *phokaina* porpoise; M.L. masc. n. *bacter* rod; M.L. masc. n. *phocoenobacter* a rod from a porpoise) [8].

The emended description of the genus includes isolates previously assigned to *P. skyensis* [4] and *Ph. atlanticus* (this study). Cells consist of Gram-negative pleomorphic rods. Non-motile and facultatively anaerobic. Blood/serum enhances growth. Colonies incubated aerobically for 48 h at 22 °C are circular, entire, low, convex, smooth, grey and<1 mm in diameter. All isolates grow aerobically between 15 and 30 °C. Growth at 4 and 37 °C is variable, with type strain NCTC 12872^T^ growing well at 37 °C but not at 4 °C. Non-haemolytic, or weakly alpha- or beta-haemolytic. Oxidase is produced. Production of catalase varies. Neither X nor V factors are required for growth. Arginine dihydrolase is negative. Indole, lysine decarboxylase and ornithine decarboxylase are variable. VP positive. Produce acid (weakly) from d(+) glucose, lactose and d(+) maltose. Acid production from d(+) cellobiose is negative. Acid production from d(−) arabinose, d(−) mannitol, d(+) mannose, d(+) sorbitol, trehalose and d(+) xylose is variable. Sensitive to the vibriostatic agent O129. Beta-galactosidase and alpha-fucosidase production is variable.

The type species is *Ph. uteri*, with NCTC 12872^T^ designated as the type strain [8]. *Phocoenobacter* is a member of the family *Pasteurellaceae* [25] and has thus far been documented exclusively in association with marine teleosts and cetaceans [7].

### Emended description of *Phocoenobacter uteri* [8]

*Phocoenobacter uteri* (ute.ri. L. masc. n. uteris uterus) [8].

The description of *Ph. uteri* is as previously described by Foster *et al*. [8], with the following modifications: originally described as catalase, lysine decarboxylase, ornithine decarboxylase, lactose and d(+) maltose negative [8]; in our hands, using a heavy inoculum method in BBL™ Phenol Red Broth Base (Becton Dickinson) supplemented with 10% foetal bovine serum (Biosera), *Ph. uteri* NCTC 12872^T^ was found positive for all. Beta-galactosidase and alpha-fucosidase are not produced.

The type strain is NCTC 12872^T^ (=M1063U/93^T^=DSM 15746^T^=CCUG 47322^T^), isolated from a harbour porpoise in Scotland. The genome assembly (2.18 Mbp; 36.17 mol% G+C content) and 16S rRNA gene sequence are available, respectively, through accession numbers GCF_900454895 and NR_027217.

## Description of *Phocoenobacter skyensis* comb. nov.

*Phocoenobacter skyensis* (skye.ensis. N.L. fem. adj. skyensis pertaining to Skye, the island in Scotland, UK, where the first two isolates of this species were isolated) [4].

Basonym: *Pasteurella skyensis* [4].

The description of *Ph. skyensis* is as previously described for *P. skyensis* [4], with the following additions and modifications: while some isolates are capable of aerobic growth at 37 °C, the type strain does not grow at this temperature. Catalase production is negative. Acid production from d(+) mannose and d(−) sorbitol is positive. Acid production from d(+) xylose is negative. Acid production from d(−) arabinose, d(−) mannitol and trehalose is variable, with type strain 95A1^T^ being arabinose and mannitol negative and trehalose positive. Beta-galactosidase is produced. Alpha-fucosidase production is variable, with the type strain being positive.

The type strain is 95A1^T^ (=DSM 24204^T^=NCIMB 13593^T^=NCTC 13204^T^), isolated from Atlantic salmon in Scotland. The genome assembly (2.26 Mbp; 34.92 mol% G+C content) and 16S rRNA gene sequence are available, respectively, through accession numbers GCF_013377295 and NR_025359.

## Description of *Phocoenobacter atlanticus* sp. nov.

*Phocoenobacter atlanticus* sp. nov. (at.lan'ti.cus. L. masc. adj. *atlanticus*, referring to the Atlantic Ocean, the known habitat of affected fish species).

Cells are Gram-negative, pleomorphic rods. Non-motile. Following 2–4 days of incubation at 22 °C on BAS, low, grey, convex, friable, non-adherent, smooth, circular colonies of≤1 mm in diameter, with an entire margin, are observed. Weak alpha-haemolysis on blood-containing agar may be observed after 7 days of aerobic incubation at 22 °C. Growth occurs at 4–30 °C, but not at 37 °C. Growth is enhanced by the supplementation of culture medium with blood and/or serum. There is a variable ability to grow on blood agar without extra NaCl added and under anaerobic conditions. X- and V-factors are not required for growth. All isolates are catalase and indole negative and oxidase and VP positive. Acid is produced from d(+) mannose. Acid production from d(−) arabinose, d(−) mannitol, d(+) sorbitol, trehalose and d(+) xylose is variable. Lysine decarboxylase and ornithine decarboxylase are not produced. Beta-galactosidase and alpha-fucosidase production is variable.

The type strain is NVIB3624^T^ (=NCIMB 15512^T^=CCUG 77520^T^), isolated from Atlantic salmon in Norway. The genome assembly (2.18 Mbp; 34.27 mol% G+C content) and 16S rRNA gene sequence are available, respectively, through accession numbers GCF_030764225 and OR265624.

## Description of *Phocoenobacter atlanticus* subspecies *atlanticus* subsp. nov.

*Phocoenobacter atlanticus* sp. nov. subspecies *atlanticus* (at.lan'ti.cus. L. masc. adj. *atlanticus*, referring to the Atlantic Ocean, the known habitat of affected fish species).

As for *Ph. atlanticus*, it is clearly facultatively anaerobic, with visible colonies after 3 days of anaerobic incubation at 22 °C. While growth is enhanced by further supplementation of NaCl, visible colonies develop on blood agar following 4 days of aerobic incubation at 22 °C. Acid is produced from d(−) arabinose, d(−) mannitol, d(+) sorbitol, trehalose and d(+) xylose. Beta-galactosidase production is variable but present in the majority (9/10) of tested isolates, including the type strain. Alpha-fucosidase is not produced.

The type strain is NVIB3624^T^ (=NCIMB 15512^T^=CCUG 77520^T^), isolated from Atlantic salmon in Norway. The genome assembly (2.18 Mbp; 34.27 mol% G+C content) and 16S rRNA gene sequence are available, respectively, through accession numbers GCF_030764225 and OR265624.

## Description of *Phocoenobacter atlanticus* subspecies *cyclopteri* subsp. nov.

*Phocoenobacter atlanticus* sp. nov. (at.lan'ti.cus. L. masc. adj. *atlanticus*, referring to the Atlantic Ocean, the known habitat of affected fish species) subsp. *cyclopteri* (cyc.lo'pte.ri. N.L. gen. n. *cyclopteri*, of *Cyclopterus*, a fish genus from which the bacterium has been commonly isolated).

As for *Ph. atlanticus*, it displays extremely poor facultative anaerobic growth, with visible growth (but not individual colonies) only after 7 days of anaerobic incubation at 22 °C. Blood agar supports slow growth, with >4 days of aerobic incubation at 22 °C required before individual colonies become visible. Acid is not produced from d(-) arabinose, d(-) mannitol, d(+) sorbitol, trehalose, or d(+) xylose. Beta-galactosidase is not produced. Alpha-fucosidase is produced.

The type strain is NVIO9100^T^ (=NCIMB 15511^T^=CCUG 77521^T^), isolated from lumpfish in Norway. The genome assembly (2.30 Mbp; 34.47 mol% G+C content) and 16S rRNA gene sequence are available, respectively, through accession numbers GCF_018343795 and KJ585696.

## Supplementary material

10.1099/ijsem.0.006729 Uncited Table S1.

## Abbreviations

ANI: average nucleotide identity
BAS: Blood agar final concentration 2% NaCl
CCUG: Culture Collection University of Gothenburg
DNA: Deoxyribonucleic acid
NaCl: Sodium Chloride
NCBI: National Center for Biotechnology Information
NCIMB: National Collection of Industrial, Food and Marine Bacteria
NCTC: National Collection of type cultures
NVIB: Norwegian Veterinary Institute Bergen
NVIO: Norwegian Veterinary Institute Oslo
RNA: Ribosomal ribonucleic acid
rpoB: gene encoding for β - subunit of bacterial RNA polymerase
16S rRNA: 16S rRNA gene
TSA: tryptic soy agar
VP: Voges–Proskauer
