# Corrigendum: Reclassification of Pasteurella skyensis as Phocoenobacter skyensis comb. nov. and description of Phocoenobacter atlanticus sp. nov. isolated from diseased Atlantic salmon (Salmo salar) and lumpfish (Cyclopterus lumpus), with subdivision into Phocoenobacter atlanticus subspecies atlanticus subsp. nov. and Phocoenobacter atlanticus subspecies cyclopteri subsp. nov.

**DOI:** 10.1099/ijsem.0.006788

**Published:** 2025-05-19

**Authors:** Hanne K. Nilsen, Anne Berit Olsen, Thomas H. Birkbeck, Farah Manji, Duncan J. Colquhoun, Snorre Gulla

**Affiliations:** 1 Section of Aquatic Biosecurity Research, Norwegian Veterinary Institute, Bergen, Norway; 2 Division of Infection and Immunity, University of Glasgow, Glasgow, Scotland, UK; 3 Mowi Norway, Bergen, Norway; 4 Fish Health Research Group, Norwegian Veterinary Institute, Ås, Norway

In the published version of this article, in Table 1, for isolate IDs NVIO3648, NVIB164, NVIB3802, NVIB543, NVIB1365, NVIB3801, NVIB1926, NVIB2702, NVIB2993 and 01A1 only, incorrect RefSeq acc. nos. were unfortunately listed in the initially published version of the table. These numbers have now been rectified in the Table 1 version accompanying this corrigendum.

**Table 1.** Isolates included in the study

Proposed species/subspecies	Isolate id.	Alternative id.	Isolated from	Year	Country	Latitude*	Longitude*	RefSeq acc. no.	Initial ref.
*Ph*. *atlanticus* subsp. *atlanticus*	NVIB3689	NVIO1993	*Salmo salar*	1991	Norway	69.649610	18.957010	GCF_030764075.1	[10]
*Ph*. *atlanticus* subsp. *atlanticus*	NVIB3131		*Salmo salar*	1999	Norway	60.200000	5.700000	GCF_030764485.1	[7]
*Ph*. *atlanticus* subsp. *atlanticus*	NVIB3694	NVIO8239	*Salmo salar*	2012	Norway	61.170833	5.293056	GCF_030764015.1	[10]
*Ph*. *atlanticus* subsp. *atlanticus*	NVIB2635		*Salmo salar*	2018	Norway	60.350000	5.000000	GCF_030764645.1	[7]
*Ph*. *atlanticus* subsp. *atlanticus*	NVIB3624^T^	NCIMB 15512^T^	*Salmo salar*	2019	Norway	61.599600	5.032800	GCF_030764225.1	[7]
*Ph*. *atlanticus* subsp. *atlanticus*	NVIB3687		*Salmo salar*	2019	Norway	62.471239	6.154239	GCF_030764125.1	[7]
*Ph*. *atlanticus* subsp. *atlanticus*	NVIB3642		*Salmo salar*	2019	Norway	61.808611	5.420833	GCF_030764205.1	[7]
*Ph*. *atlanticus* subsp. *atlanticus*	NVIB3672		*Salmo salar*	2019	Norway	60.370000	6.143889	GCF_030764105.1	[7]
*Ph*. *atlanticus* subsp. *atlanticus*	NVIB3763		*Salmo salar*	2020	Norway	60.350000	5.000000	GCF_030763825.1	[7]
*Ph*. *atlanticus* subsp. *atlanticus*	NVIB3708		*Salmo salar*	2020	Norway	60.037778	5.268333	GCF_030763925.1	[7]
*Ph*. *atlanticus* subsp. *cyclopteri*	NVIO3648		*Cyclopterus lumpus*	1996	Norway	69.649610	18.957010	GCF_030763365.1	[7]
*Ph*. *atlanticus* subsp. *cyclopteri*	NVIB164		*Cyclopterus lumpus*	2012	Norway	58.970000	5.731389	GCF_030764725.1	[7]
*Ph*. *atlanticus* subsp. *cyclopteri*	NVIB3802		*Cyclopterus lumpus*	2013	Norway	68.221389	13.784444	GCF_030763465.1	[7]
*Ph*. *atlanticus* subsp. *cyclopteri*	NVIB543	NVIO9294	*Cyclopterus lumpus*	2013	Norway	58.970000	5.731389	GCF_030763415.1	[10]
*Ph*. *atlanticus* subsp. *cyclopteri*	NVIO9100^T^	NCIMB 15511^T^	*Cyclopterus lumpus*	2013	Norway	62.567500	6.372222	GCF_018343795.1	[10]
*Ph*. *atlanticus* subsp. *cyclopteri*	NVIB1365		*Cyclopterus lumpus*	2016	Norway	60.402778	5.805556	GCF_030764785.1	[7]
*Ph*. *atlanticus* subsp. *cyclopteri*	NVIB3801		*Cyclopterus lumpus*	2017	Norway	69.300000	17.666667	GCF_030763505.1	[7]
*Ph*. *atlanticus* subsp. *cyclopteri*	NVIB1926		*Cyclopterus lumpus*	2017	Norway	60.200000	5.700000	GCF_030764685.1	[7]
*Ph*. *atlanticus* subsp. *cyclopteri*	NVIB2702		*Cyclopterus lumpus*	2018	Norway	60.200000	5.700000	GCF_030764665.1	[7]
*Ph*. *atlanticus* subsp. *cyclopteri*	NVIB2993		*Cyclopterus lumpus*	2018	Norway	59.998333	5.577222	GCF_030764525.1	[7]
*Ph*. *skyensis*	95 A1^T^	NCIMB 13593^T^	*Salmo salar*	1995	Scotland	56.659406	−4.011214	GCF_013377295.1	[4]
*Ph*. *skyensis*	01 A1		*Salmo salar*	2001	Scotland	56.659406	−4.011214	GCF_030765025.1	[6]
*Ph*. *skyensis*	TW34_18		*Salmo salar*	2017	Scotland	56.659406	−4.011214	GCF_030764825.1	[7]
*Ph*. *skyensis*	TW260_19		*Salmo salar*	2019	Scotland	56.659406	−4.011214	GCF_030764885.1	[7]
*Ph*. *skyensis*	NVIO11850		*Salmo salar*	2020	Norway	60.066667	6.550000	GCF_030763395.1	[9]
*Ph*. *uteri*	NCTC 12872^T^	M1063U/93^T^	*Phocoena phocoena*	1993	Scotland	56.659406	−4.011214	GCF_900454895.1	[8]

*Approximate coordinates (municipal administrative centre or midpoint) are provided in order to retain the anonymity of affected aquaculture sites in Norway. For Scottish isolates, available information on their geographic origins was limited.

The authors apologise for any inconvenience caused.

